# Surgical Extent of Central Lymph Node Dissection for Papillary Thyroid Carcinoma Located in the Isthmus: A Propensity Scoring Matched Study

**DOI:** 10.3389/fendo.2021.620147

**Published:** 2021-06-15

**Authors:** Yanjie Shuai, Kai Yue, Yuansheng Duan, Mengqian Zhou, Yan Fang, Jin Liu, Dandan Liu, Chao Jing, Yansheng Wu, Xudong Wang

**Affiliations:** Department of Maxillofacial & E.N.T Oncology, Tianjin Medical University Cancer Institute & Hospital, Key Laboratory of Cancer Prevention and Therapy, Tianjin Cancer Institute, National Clinical Research Center of Cancer, Tianjin, China

**Keywords:** isthmic papillary thyroid carcinoma, propensity score matching, central lymph node metastasis, central lymph node dissection, recurrence, survival

## Abstract

**Introduction:**

To assess the risk factor for the central lymph node (CLN) metastasis and investigated the surgery extent of lymph node dissection for patients with isthmic PTC (papillary thyroid carcinoma).

**Materials and Methods:**

A total of 669 patients with a single nodule of isthmic PTC were retrospectively reviewed. The propensity score matching was performed twice separately. 176 patients respectively from patients who underwent thyroidectomy plus bilateral central lymph node dissection (BCLND) and who underwent thyroidectomy plus unilateral central lymph node dissection (UCLND) were matched. 77 patients were respectively selected from patients who underwent thyroidectomy plus BCLND and who underwent thyroidectomy with no central lymph node dissection (NCLND) were matched.

**Results:**

Among all the patients who underwent BCLND, 81/177 (45.76%) was confirmed with histologically positive CLN metastasis, and the occult lymph node metastasis is 25.42%. A tumor size of 1.05 cm was calculated as the cutoff point for predicting CLN metastasis by ROC curve analysis with 177 patients who underwent BCLND. The 5-year recurrence-free survival (RFS) rates were 92.9% in the NCLND group and 100% in the BCLND group with *P*<0.05, while there was no statistical difference in 5-year RFS between the BCLND group and UCLND group (*P*=0.11). The multivariate logistic regression analysis identified that age<55, tumor size>1cm, capsule invasion and lymphovascular invasion were significantly associated with CLN metastasis, while only age and lymphovascular invasion were proved to be independent risk factors related to contralateral CLN metastasis.

**Conclusions:**

The thyroidectomy with NCLND could be insufficient for patients with isthmic PTC especially for those patients with high risk of central lymph node metastasis, considering that the rate of occult lymph node metastasis could not be ignored.

## Introduction

Papillary thyroid carcinoma (PTC) is the most common type of differentiated thyroid carcinoma, approximately accounting for 90% of all thyroid carcinomas and its incidence increased annually (1, 2). PTC is known to be associated with good prognosis with overall disease-specific mortality rates<10% at 20-year follow-up (3). However, high frequency of cervical lymph node metastasis had been regarded as the characteristic of PTC, which could be associated with the local recurrence and distant metastasis (4, 5). Previous studies reported that the tumor originating in the isthmus of thyroid might have a greater tendency to central lymph nodes (CLN) metastasis, especially to pretracheal and bilateral paratracheal lymph nodes (6–8).

In addition, a series of studies have associated isthmic PTC with multiple foci, local invasion to adjacent tissues and the high rate of bilateral CLN metastasis (9). A total thyroidectomy was recommended for patients with a single tumor nodule if the tumor diameter is larger than 4cm, or with extrathyroidal extension, known distant metastases or cervical lymph node metastases, or prior radiation exposure according to the National Comprehensive Cancer Network (NCCN) guideline (10). However, different opinions about the prophylactic lymph nodes dissection still exist in the previous studies (11–13), and the extent of CLN dissection has not reached a consensus. The current guidelines have not provided a suggested surgical treatment for the isthmic PTC (14). Therefore, in the present study, we aim to assess the risk factor for the CLN metastasis and investigate the surgery extent of lymph nodes dissection for patients with PTC arising from isthmus.

## Materials and Methods

### Patients

We retrospectively reviewed the medical records of 723 patients with a single lesion at the location of isthmus of thyroid, who undergone surgical treatment at Tianjin Medical University Cancer Institute and Hospital between January 2012 and December 2018 and were pathologically diagnosed with PTC. All of the patients involved in the study underwent thyroidectomy, including total thyroidectomy, lobectomy + isthmusectomy, or isthmusectomy. 51 patients were excluded for undergoing lateral neck lymph node dissection, 3 patients were excluded for losing follow-up. From a total of 669 patients, 177 patients underwent thyroidectomy plus bilateral central lymph node dissection (BCLND), 401 patients underwent thyroidectomy plus unilateral central lymph node dissection (UCLND), and 91 patients underwent thyroidectomy with no central lymph node dissection (NCLND). The inclusion criteria were as follows: (1) patients was primarily diagnosed with a single tumor nodule located in thyroid isthmus and underwent thyroid surgical treatment, (2) patients was pathologically diagnosed with PTC, (3) patients did not have lateral neck lymph node metastasis, (4) patients did not have a history of cancer disease or treatment, or a history of neck radiotherapy, and (5) patients had complete clinical, pathological and follow-up data. This retrospective study was approved by the Clinical Research Ethics Committee of Tianjin Medical University Cancer Institute and Hospital. Written-informed consent was obtained from all patients.

All patients involved underwent preoperative physical examination and imaging examination. Ultrasound was performed to evaluate the location of tumor nodule and central lymph node metastasis. Ultrasonography guided fine-needle aspiration cytology (FNAC) and enhanced CT was performed when the lateral lymph nodes were suspicious for metastasis. Isthmic PTC was defined as a tumor nodule located in the central part of thyroid gland lying anterior to the trachea, overlying the second to fourth tracheal rings according to ultrasonography. The tumor was classified as left isthmus or right isthmus based on the ultrasonography and the recorded intraoperative data.

Baseline clinicopathological information of each patient, including preoperative clinical and radiological evaluations, pathological examinations, patterns of lymph node metastasis, and follow-up data were collected. The surgical extent was decided by the surgeon based on the reference and the will of patients. All of the operations were performed by surgeons with at least 20 years of thyroid surgery experience, and central lymph node dissection was performed strictly following the specifications in the American Thyroid Association guidelines (14). Recurrence was defined as any new lesions confirmed with cytologic or pathological examination after surgery. The median follow-up time was 53 months (range from 14 to 96 months), and no patient died from tumor recurrence or distant metastasis.

### Propensity Score Matching Analysis

**Figure 1:**
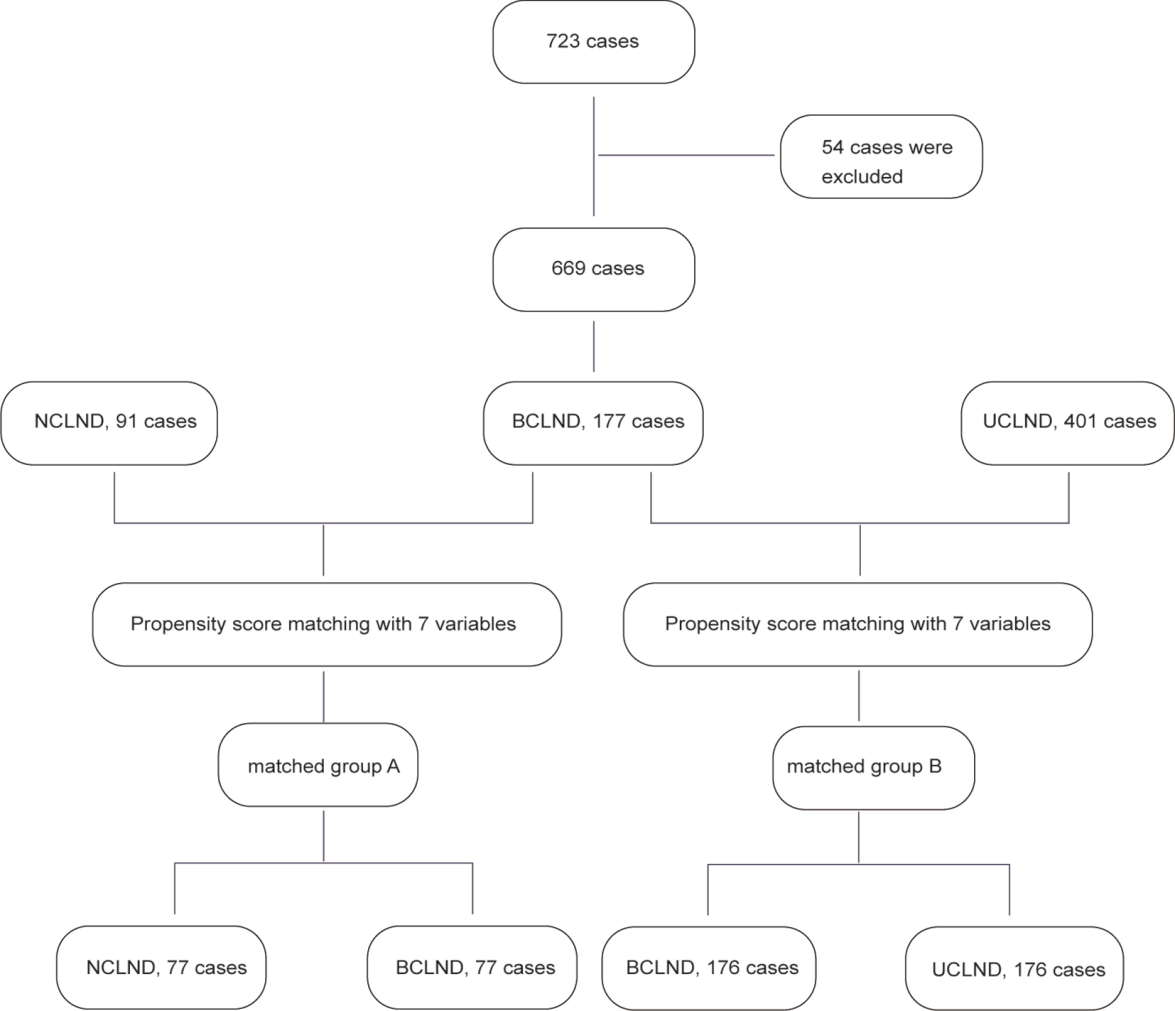
Flowchart of patients with isthmic papillary thyroid cancer in this study.

Propensity score matching was used to effectively reduce the selection bias and confounding differences. Seven variables including sex, age, tumor location, tumor size, capsule invasion, lymphovascular invasion, and lymph node metastasis shown by preoperative ultrasound were analyzed using the method of propensity score matching. We constructed one matched group through propensity score matching analysis between patients who underwent thyroidectomy plus BCLND and patients who underwent thyroidectomy plus UCLND. Another matched group was established between patients who underwent thyroidectomy plus BCLND and patients who underwent thyroidectomy alone using propensity score matching. The recurrence-free survival (RFS) was compared in the matched groups (**Figure 1**).

### Statistical Analysis

Statistical analysis was performed using SPSS version 25 (SPSS, Inc. Chicago, IL, USA) and R software version (3.6.1), Baseline clinical characteristics were compared using t test for continuous variables and chi-square test for categorical variables. Kaplan–Meier survival analysis and log-rank test were used to estimate the RFS. A receiver operating characteristic (ROC) curve analysis was used to identify the cutoff point of tumor size for predicting the risk of CLN metastasis. Univariate and multivariate analyses were performed with the logistic regression model. *P* < 0.05 was considered statistically significant.

## Results

### Baseline Characteristics and Propensity Score Matching

Of all the 669 patients, most of the patients were female (493/669, 73.69%). The rate of capsular invasion of the patients enrolled in the study was 59.64%. Among the patients underwent BCLND, 81/177 (45.76%) was confirmed with histologically positive CLN metastasis after surgery, and the occult lymph node metastasis is 25.42%. Of all the patients who underwent UCLND, 152/401(37.91%) was defined as histologically positive CLN metastasis after surgery.

Propensity score matching was performed to generate the matched groups. 176 patients from 401 patients who received UCLND and 176 patients from 177 patients who received BCLND were matched. Similarly, 77 patients from 177 patients who accepted BCLND and 77 patients from 91 patients who have not received lymph node dissection were compared. The clinicopathological characteristics after propensity score matching were presented respectively in the **Table 1** and **Table 2**. The median tumor size was respectively 0.92 ± 0.47 and 0.89 ± 0.56 in the matched BCLND group and NCLND group, with *P*=0.663. No significant difference exists in the median tumor size between the matched BCLND group and UCLND group (1.05 ± 0.55 *vs* 0.98 ± 0.64, *P*=0.259). Similarly, there was no significant difference observed in age, sex, tumor location, capsular invasion, lymphovascular invasion, and lymph node metastasis shown by preoperative ultrasound after propensity score matching between the matched BCLND group and UCLND group, as well as the matched BCLND group and NCLND group. The median follow-up time of all of the involved patients is 53 months (range:14-96 months, mean:56 months). The median follow-up time of patients who underwent BCLND and patients who underwent NCLND in the matched group A is respectively 62 months (range:14-96 months, mean:58.5 months) and 57 (range:20-96, mean:58.6). The median follow-up time of patients who underwent BCLND and patients who underwent UCLND in the match group B is respectively 50 months (range:14-96 months, mean:53.8 months) and 34 months (range:14-92 months, mean:35.5 months).

**Table 1:** Baseline clinicopathological characteristics of patients with PTC located at isthmus with BCLND or UCLND after propensity score matching.

Variables	BCLND (n = 176)	UCLND (n = 176)	*P*
Sex			
Female	138 (78.4%)	132 (75.0%)	0.449
Male	38 (21.6%)	44 (25.0%)	
Age, years	47.18 ± 10.92	45.49 ± 9.89	0.129
Tumor size, cm	1.05 ± 0.55	0.98 ± 0.64	0.259
Location			
Left isthmus	96 (54.5%)	92 (51.4%)	0.669
Right isthmus	80 (45.5%)	84 (48.6%)	
Capsular invasion			
No	51 (29.0%)	38 (21.6%)	0.111
Yes	125 (71.0%)	138 (78.4%)	
Lymphovascular invasion			0.214
No	165 (93.8%)	170 (96.6%)	
Yes	11 (6.3%)	6 (3.4%)	
Hashimoto thyroiditis			0.342
No	139 (79.0%)	146 (83.0%)	
Yes	37 (21.0%)	30 (17.0%)	
lymph node metastasis detected by preoperative ultrasound			0.794
No	138 (78.4%)	140 (79.5%)	
Yes	38 (21.6%)	36 (20.5%)	

**Table 2:** Baseline clinicopathological characteristics of patients with PTC located at isthmus with BCLND or NCLND after propensity score matching.

Variables	BCLND (n = 77)	NCLND (n = 77)	*P*
Sex			
Female	61 (79.2%)	61 (79.2%)	1.000
Male	16 (20.8%)	16 (20.8%)	
Age, years	46.99 ± 11.18	46.81 ± 10.08	0.916
Tumor size, cm	0.92 ± 0.47	0.89 ± 0.56	0.663
Location			
Left isthmus	35 (45.5%)	36 (46.8%)	0.872
Right isthmus	42 (54.5%)	41 (53.2%)	
Capsular invasion			
No	35 (45.5%)	40 (51.9%)	0.420
Yes	42 (54.5%)	37 (48.1%)	
Lymphovascular invasion			1.000
No	72 (93.5%)	73 (96.6%)	
Yes	5 (6.5%)	4 (5.2%)	
Hashimoto thyroiditis			0.338
No	69 (89.6%)	65 (84.4%)	
Yes	8 (10.4%)	12 (15.6%)	
lymph node metastasis detected by preoperative ultrasound			–
No	77 (100%)	77 (100%)	
Yes	0 (0%)	0 (0%)	

### Postoperative Tumor Recurrence

**Figure 2:**
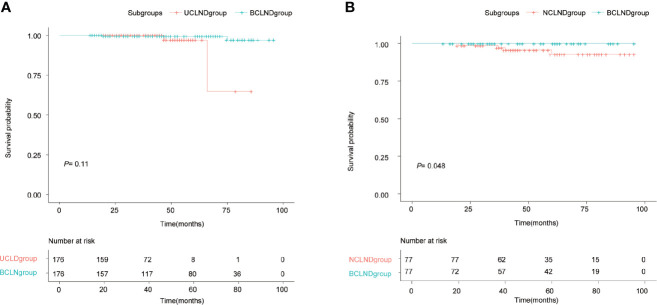
Kaplan-Meier recurrence-free survival curves. **(A)** Kaplan-Meier recurrence-free survival curve of patients with isthmus PTC who underwent thyroidectomy with bilateral central neck dissection (BCLND) and who underwent thyroidectomy with unilateral central neck dissection (UCLND) (*P*=0.11). **(B)** Kaplan-Meier recurrence-free survival curve of patients with isthmus PTC who underwent thyroidectomy with bilateral central neck dissection (BCLND) and who underwent thyroidectomy with no central neck dissection (NCLND) (*P*=0.048).

In the matched group, recurrence occurred in 2 cases of the BCLND group and 2 cases of the UCLND group. The 5-year RFS rates were respectively 97.1% in the UCLND group and 99.4% in the BCLND group. Kaplan-Meier curves showed no statistically difference in the 5-year RFS between the BCLND group and UCLND group (*P* = 0.11) (**Figure 2A**). In the other matched group, the 5-year RFS rates were 92.9% in the NCLND group and 100% in the BCLND group, with statistically significant (*P* = 0.048) (**Figure 2B**).

### Risk Factors for CLN Metastasis

**Figure 3:**
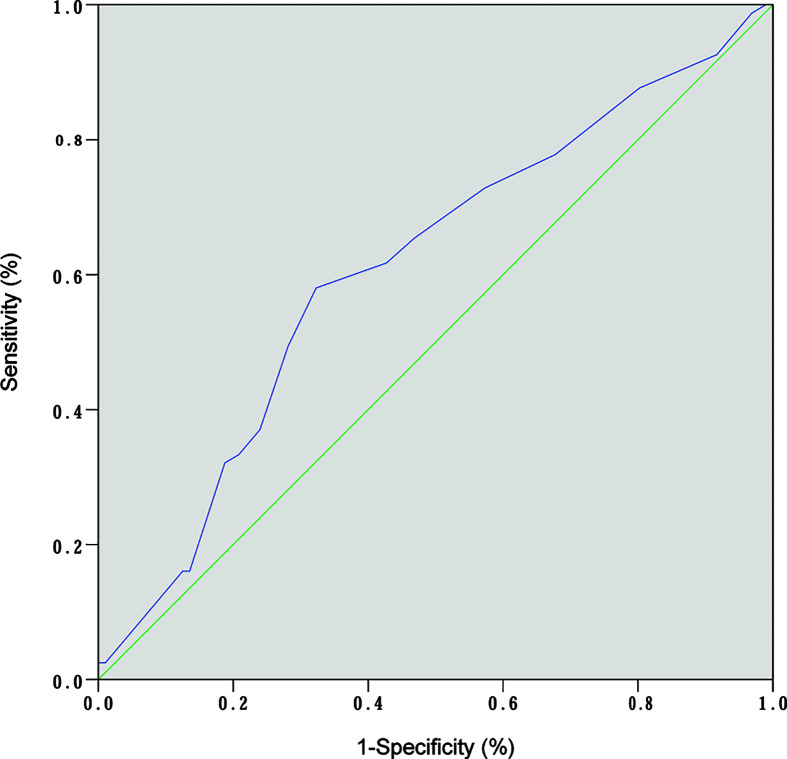
ROC curve analysis of tumor size and CLN metastasis. (AUC:0.610; 95% CI: 0.526–0.694; *P*= 0.01).

**Figure 4:**
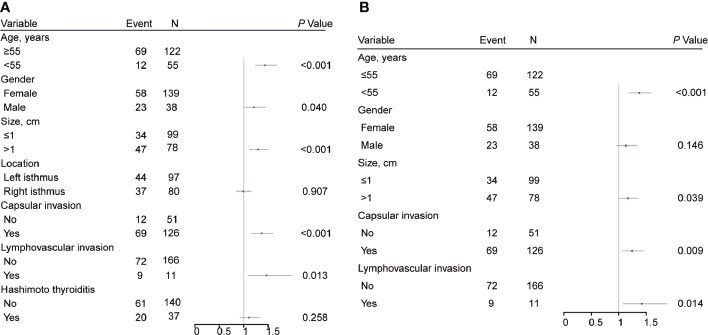
**(A)** Univariate logistic regression analysis of clinicopathological factors associated with CLN metastasis for patients underwent thyroidectomy with BCLND. **(B)** Multivariate logistic regression analysis of clinicopathological factors associated with CLN metastasis for patients underwent thyroidectomy with BCLND.

**Figure 5:**
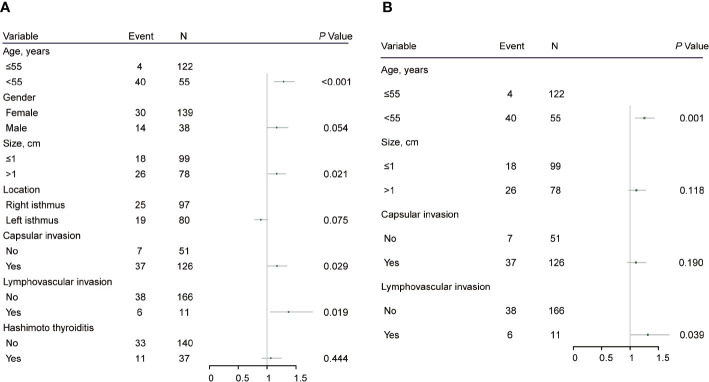
**(A)** Univariate logistic regression analysis of clinicopathological factors associated with contralateral CLN metastasis for patients underwent thyroidectomy with BCLND. **(B)** Multivariate logistic regression analysis of clinicopathological factors associated with contralateral CLN metastasis for patients underwent thyroidectomy with BCLND.

ROC curve analysis was performed to make out the cutoff point of tumor size for predicting CLN metastasis in 177 patients, who underwent the BCLND. According to the ROC curve analysis, the AUC of the model for prediction of CLN metastasis was 0.610 (95% CI: 0.526–0.694; *P*=0.012). The analysis indicated that a tumor size of 1.05 cm was the cutoff point for predicting CLN metastasis (**Figure 3**). 45.76% (81/177) was confirmed with histologically positive CLN metastasis. Of them, 24.9% (44/177) patients were diagnosed with contralateral CLN metastasis. In the univariate logistic regression, male (OR:1.21; 95% CI, 1.01–1.44; *P*=0.04), age<55 (OR:1.44; 95% CI:1.24–1.68; *P*<0.001), tumor size>1 (OR:1.30; 95% CI:1.12–1.50; *P*<0.001), capsule invasion (OR:1.37; 95% CI:1.17–1.60; *P*<0.001) and lymphovascular invasion (OR:1.47; 95% CI:1.09–1.98; *P*=0.013) were significantly related to CLN metastasis (**Figure 4A**). To exclude the possibility that clinical and pathological factors might bias CLN metastasis, factors shown to be significant in the univariate analysis were involved into the multivariate logistic regression analysis. The multivariate logistic regression analysis showed that age<55 (OR:1.37; 95% CI:1.19–1.58; *P*<0.001), tumor size>1 cm (OR:1.17; 95% CI:1.01–1.35; *P*= 0.039), capsule invasion (OR:1.24; 95% CI:1.06–1.46; *P*= 0.009) and lymphovascular invasion (OR:1.42; 95% CI:1.08–1.87; *P*=0.014) were significantly associated with CLN metastasis (**Figure 4B**). In the univariate logistic regression analysis, age<55 (OR:1.29; 95% CI:1.12–1.47; *P*<0.001), tumor size>1 (OR:1.16; 95% CI:1.02–1.32; *P*=0.021), capsule invasion (OR:1.17; 95% CI:1.02–1.34; *P*=0.029) and lymphovascular invasion (OR:1.37; 95% CI:1.06–1.78; *P*= 0.019) were statistically related to contralateral CLN metastasis (**Figure 5A**). While the multivariate logistic regression analysis identified that only age<55 (OR:1.25; 95% CI:1.10–1.43; *P*=0.001) and lymphovascular invasion (OR:1.31; 95% CI:1.02–1.69; *P=*0.039) were independent risk factors for contralateral CLN metastasis (**Figure 5B**).

## Discussion

Thyroid cancer is more common arising in the left or right lobes of thyroid gland. The incidence of thyroid cancer located at isthmus is relatively low, only about 1% to 10% occurred according to reports (15). The isthmus is the central part of thyroid gland that connects the right and left thyroid lobes, and it is composed of thin (2–6 mm) and small-volume thyroid parenchyma. As a result of its superficial anatomic location, nodules limited to the isthmus were more easily detected. In addition, with the improvement of diagnostic procedures, most of the tumor diameter is small when it is detected.

Nevertheless, PTC confined to isthmus has been reported to be more aggressive than the nodule located at lobes (15–17). Young Chan Lee et al. (18) considered that isthmic PTC was more likely to be accompanied by capsule invasion and the pretracheal or prelaryngeal lymph nodes metastasis, being consistent with the other studies reported (19, 20). The invasive clinicopathological features of isthmic PTC might be attributable to the thin shape of the isthmus, thus facilitating the invasion of adjacent tissues. In our study, the rate of capsular invasion was 59.64%, significantly higher than a meta-analysis reported 21.82% in PTC originating in the lobe (21). The incidence of capsular invasion (59.64%) that our data obtained was slightly lower than that reported by Seung Taek Lim et al. (63.7%) (22) and Lee et al. (70.2%) (23). That might be owing to the patients, most of whom included in our study were diagnosed in relatively early-stage.

Currently, the role of prophylactic central lymph node dissection (CLND) in preventing the nodal recurrence of PTC remains controversial (5, 24). The thorough resection of central lymphatic tissue can reduce the risk of recurrence by eliminating potential lymph node metastasis (25, 26). However, the intraoperative exploration may make an injure on the parathyroid glands and recurrent laryngeal nerve, even it is of small chance, it still has a great impact on the living quality of patients when it occurs. Hence, it is of great importance to investigate a suitable surgical method and explore the risk factors of lymph node metastasis for isthmic PTC. Although many studies have made efforts on the surgical extent of isthmic PTC, most of which involved a relatively small simple size of patients. In this study, a larger size of patients with isthmic PTC who underwent multiple surgical methods were incorporated. Moreover, we analyzed the influence of three different lymph node dissection extent of surgical methods on RFS compared with other studies that usually included two surgical procedures. To eliminate the bias, propensity score method was performed. Propensity score method is increasingly being applied to evaluate the effects of various therapies. The propensity score matching analysis was employed for balancing the baseline characteristics to better evaluate the effect of different extent of lymph node dissection for isthmic PTC in this study.

As a result of the special anatomical location, the lymphatic drainage mechanism of isthmus is unclear. CLN metastasis is usually observed when patients are firstly diagnosed with PTC, with reported incidences from 20% to 39% (27, 28). Previous studies showed that patients with isthmic PTC tended to be more prone to get CLN metastasis (7–9, 18, 29). A study reported that the rate of CLN metastasis of isthmic PTC was 63.8%, higher than that of non-isthmus PTC (40.3%) with *P* < 0.001 (23), and also higher than 29.6% that reported in a previous study (30). Young Woo Chang et al. (31) also reported that the isthmic PTC had an increased frequency of bilateral CLN metastasis than the tumor located in lobe, indicating a higher rate of contralateral node metastasis in the isthmic PTC. In this study, among the patients underwent BCLND, 81/177 (45.76%) was confirmed with histologically positive CLN metastasis. A possible explanation for the higher frequency of CLN metastasis seems to be due to the midline position of isthmus tumors, which are able to spread into both paratracheal lymph nodes. Therefore, some investigators considered that total thyroidectomy with BCLND should be regarded as the standard surgical protocol for PTC located in isthmus of thyroid (29, 31). However, a research involving 27 patients who underwent isthmusectomy or isthmusectomy with unilateral lobectomy, showed that a 5-year RFS of 95.2% and suggested that an isthmusectomy or extended isthmusectomy could be feasible for patients with well different thyroid carcinoma confined to the isthmus. Similarly, Hakyoung Park et al. (12) investigated the outcomes of 43 patients treated with isthmusectomy alone for PTC during a 30-year period and summarize that isthmusectomy is considered adequate in low-risk patients with PTC arising in isthmus, excepting for patients with the risk factors such as extrathyroid extension, CLN metastasis, or with other nodules in the thyroid lobes.

Our study used propensity score matching method to balance the bias that could be generated from sex, age, tumor size or the lymph node metastasis status shown by preoperative ultrasound. Thus, the selection bias could be ignored when the different extent of lymph node dissection was compared. The 5-year RFS of patients who received BCLND was better than that of patients who underwent NCLND, which indicated that the surgery method without lymph nodes dissection may be insufficient for patients with isthmic PTC. Additionally, the results indicated that the RFS did not differ between the BCLND group and UCLND group. However, due to the median follow-up time of patients of UCLND group in the matched group B is 34 months, which might make the result less reliable. In addition, in the multivariate logistic regression analysis, age<55 and lymphovascular invasion were independent risk factors associated with contralateral CLN metastasis. Therefore, BCLND should be considered to be performed on patients with risk factors for contralateral CLN metastasis, such as age<55 and lymphovascular invasion, even there was no statistical difference in the 5-year RFS between the BCLND group and UCLND group. A multicenter retrospective research reported (32) that age<45 and tumor size>5 mm were related to CLN metastasis. Another research revealed (9) that age<45, minimal extrathyroidal extension and lymphovascular invasion were associated with CLN metastasis. According to our results, age<55, tumor size>1 cm, capsule invasion and lymphovascular invasion were risk factors associated with CLN metastasis. So that, at least UCLND should also be considered for patients with these risk factors. What is more, the choice about the surgical extent should be made taking the preoperative occult lymph nodes metastasis into account.

The reported occult metastasis rate of PTC was about 27% to 90% (29, 33–35). In our study, we reviewed the preoperative ultrasonographic reports of all patients who underwent BCLND, and found that the rate of occult lymph node metastasis is 25.42%. Even though our result is lower than previous studies reported, it is still a high rate that we could not ignore. The high rate of occult metastasis was mainly because of the air disturbance from the trachea when the preoperative ultrasonography was used for determining CLN metastasis, thus resulting in the lower accuracy of diagnosis.

Thyroidectomy with CLND is a mature surgical approach that usually being used in the clinical practice. However, the ultrasound diagnosis of CLN metastasis is relatively difficult, and the anatomical location of the CLN is so special that the FNAC need to be operated by experienced doctors for the diagnosis of CLN metastasis. The result of which could be accompanied with the possibility of false-negative rates. Therefore, prophylactic central neck dissection is necessary for patients with high risk factors for CLN metastasis. In addition, some previous studies have also demonstrated that prophylactic central neck dissection could significantly reduce locoregional recurrence (36, 37).

There are several limitations in our study. Firstly, it is retrospectively designed, although the propensity score matching was employed in our study, it still cannot replace randomization process because of the unobserved values being existed. Secondly, the occurrence rate of PTC located in isthmus was relatively low, a large number of cases from multicenter could help getting more objective results. Finally, the absence of long-term follow-up could also have limited the study. Especially the median follow-up time of patients of UCLND group in the matched group B is not long enough, which could make the results less reliable. Therefore, further prospective studies with longer follow-up time, more complete data are necessary to overcome the limitations of this study.

## Conclusion

Age<55, tumor size>1cm, capsule invasion and lymphovascular invasion could be related to CLN metastasis, while age<55 and lymphovascular invasion were independent risk factors associated with contralateral CLN metastasis for isthmic PTC. Considering our results above, the thyroidectomy without lymph nodes dissection is insufficient for patients with isthmic PTC, especially for those patients with high risk of central lymph node metastasis. UCLND should be underwent at least for patients with isthmic PTC. BCLND should be considered for patients with risk factors for CLN metastasis.

## Data Availability Statement

The raw data supporting the conclusions of this article will be made available by the authors, without undue reservation.

## Ethics Statement

The study was approved by the Clinical Research Ethics Committee of Tianjin Medical University Cancer Institute and Hospital. Written informed consent was obtained from all patients. The patients/participants provided their written informed consent to participate in this study.

## Author Contributions

YS, XW, CJ, and YW conceived of and designed the research. YS edited this manuscript and participated the data collection and statistical analysis. KY and YD participated the data collection, statistical analysis, and manuscript preparation. MZ, JL, YF, and DL participated in the data acquisition. CJ, YW, and XW guided the statistical analysis and revised the manuscript. CJ, YW, and XW had primary responsibility for final content. All authors contributed to the article and approved the submitted version.

## Funding

This study was supported by National Key R&D Program of China (2019YFC0119205) and the Tianjin Science and Technology Plan Project (17YFZCSY00690).

## Conflict of Interest

The authors declare that the research was conducted in the absence of any commercial or financial relationships that could be construed as a potential conflict of interest.
