# Supplementary material for: Surgical Extent of Central Lymph Node Dissection for Papillary Thyroid Carcinoma Located in the Isthmus: A Propensity Scoring Matched Study
Source: Front Endocrinol (Lausanne). 2021 Jun 15;12:620147. doi: 10.3389/fendo.2021.620147 (PMC8240638; doi:10.3389/fendo.2021.620147)
Supplement: Supplementary file 1 [file DataSheet_1.docx]

|  | Univariate analysis | | Multivariate analysis | |
| --- | --- | --- | --- | --- |
| Variables | Odd Ratio (95%CI) | *P* | Odd Ratio (95%CI) | *P* |
| Age<55 years | 1.44 (1.24−1.68) | <0.001 | 1.37(1.19−1.58) | <0.001 |
| Male | 1.21 (1.01−1.44) | 0.040 | 1.13 (0.96−1.33) | 0.146 |
| Size>1cm | 1.30(1.12−1.50) | <0.001 | 1.17(1.01−1.35) | 0.039 |
| PTC in right isthmus | 0.99 (0.85−1.15) | 0.907 | -- | -- |
| Capsular invasion | 1.37 (1.17−1.60) | <0.001 | 1.24 (1.06−1.46) | 0.009 |
| Lymphovascular invasion | 1.47 (1.09−1.98) | 0.013 | 1.42 (1.08−1.87) | 0.014 |
| Hashimoto thyroiditis | 1.11(0.93−1.33) | 0.258 | -- | -- |

**Table S1.** OR (95%CI) of univariate and multivariate logistic regression analysis of clinicopathological factors associated with CLN metastasis for patients underwent thyroidectomy with BCLND

|  | Univariate analysis | | Multivariate analysis | |
| --- | --- | --- | --- | --- |
| Variables | Odd Ratio (95%CI) | *P* | Odd Ratio (95%CI) | *P* |
| Age<55 years | 1.29 (1.12−1.47) | <0.001 | 1.25(1.10−1.43) | 0.001 |
| Male | 1.16 (1.00−1.36) | 0.054 | -- | -- |
| Size>1cm | 1.16(1.02−1.32) | 0.021 | 1.11(0.97−1.27) | 0.118 |
| PTC in right isthmus | 0.89(0.78−1.01) | 0.075 | -- | -- |
| Capsular invasion | 1.17 (1.02−1.34) | 0.029 | 1.10 (0.95−1.28) | 0.190 |
| Lymphovascular invasion | 1.37 (1.06−1.78) | 0.019 | 1.31 (1.02−1.69) | 0.039 |
| Hashimoto thyroiditis | 1.06(0.91−1.24) | 0.444 | -- | -- |

**Table S2.** OR (95%CI) of univariate and multivariate logistic regression analysis of clinicopathological factors associated with contralateral CLN metastasis for patients underwent thyroidectomy with BCLND
